# Physical Activity in Cancer Survivors During “Re-Entry” Following Cancer Treatment

**DOI:** 10.5888/pcd15.170277

**Published:** 2018-05-24

**Authors:** Alyssa N. Troeschel, Corinne R. Leach, Kerem Shuval, Kevin D. Stein, Alpa V. Patel

**Affiliations:** 1 Intramural Research Department, American Cancer Society, Atlanta, Georgia

## Abstract

**Introduction:**

The transition from active cancer treatment into survivorship, known as *re-entry*, remains understudied. During re-entry, clinicians can educate survivors on the benefits of healthy behaviors, including physical activity, as survivors adjust to life after cancer. We examine the prevalence of adherence to established aerobic physical activity guidelines (≥150 minutes of moderate-intensity physical activity per week) in addition to related medico-demographic factors among cancer survivors during re-entry.

**Methods:**

Data from 1,160 breast, colorectal, and prostate cancer survivors participating in the American Cancer Society’s National Cancer Survivor Transition Study were examined. Multinomial logistic regression was used to calculate adjusted odds ratios (AOR) for various medico-demographic variables in relation to 4 established levels of physical activity (inactive, insufficiently active, 1–<2 times the guideline level, and ≥2 times the guideline level [referent group]).

**Results:**

Overall, 8.1% were inactive, 34.1% were insufficiently active, 24.3% were within 1 to less than 2 times the guidelines, and 33.4% exceeded guidelines by 2 or more times. Inactive people had significantly higher odds of being women (AOR, 1.88; 95% confidence interval [CI], 1.10–3.23) and having lower education levels (AOR, 2.02; 95% CI, 1.21–3.38) compared with those who exceeded guidelines by 2 or more times. Each additional comorbidity was associated with a 26% increase in odds of inactivity (AOR, 1.26; 95% CI, 1.08–1.47).

**Conclusion:**

Patient education on the benefits of regular physical activity is important for all cancer survivors and may be especially important to review after treatment completion to promote healthy habits during this transition period. Survivors who are women, are less educated, and have comorbid conditions may be less likely to be compliant with physical activity guidelines.

**MEDSCAPE CME**

Medscape, LLC is pleased to provide online continuing medical education (CME) for this journal article, allowing clinicians the opportunity to earn CME credit.

In support of improving patient care, this activity has been planned and implemented by Medscape, LLC and Preventing Chronic Disease. Medscape, LLC is jointly accredited by the Accreditation Council for Continuing Medical Education (ACCME), the Accreditation Council for Pharmacy Education (ACPE), and the American Nurses Credentialing Center (ANCC), to provide continuing education for the healthcare team.

Medscape, LLC designates this Journal-based CME activity for a maximum of 1.00 *AMA PRA Category 1 Credit(s)™*. Physicians should claim only the credit commensurate with the extent of their participation in the activity.

All other clinicians completing this activity will be issued a certificate of participation. To participate in this journal CME activity: (1) review the learning objectives and author disclosures; (2) study the education content; (3) take the post-test with a 75% minimum passing score and complete the evaluation at http://www.medscape.org/journal/pcd; (4) view/print certificate.

**Release date: May 24, 2018; Expiration date: May 24, 2019**

**Learning Objectives**

Upon completion of this activity, participants will be able to:

1. Assess the background of physical activity after cancer treatment
2. Distinguish rates of insufficient physical activity among cancer survivors in the current study
3. Evaluate sociodemographic variables associated with physical inactivity among cancer survivors
4. Review disease variables associated with physical inactivity among cancer survivors

**EDITOR**

Caran Wilbanks

Editor, *Preventing Chronic Disease*

Disclosure: Caran Wilbanks has disclosed the following relevant financial relationships:

Other: Partner is employed by Allscripts, Inc.

**CME AUTHOR**

Charles P. Vega, MD, FAAFP

Health Sciences Clinical Professor, University of California, Irvine, Department of Family Medicine; Associate Dean for Diversity and Inclusion, University of California, Irvine, School of Medicine; Executive Director, University of California, Irvine, Program in Medical Education for the Latino Community, Irvine, California

Disclosure: Charles P. Vega, MD, FAAFP, has disclosed the following relevant financial relationships:

Served as an advisor or consultant for: Johnson & Johnson Pharmaceutical Research & Development, L.L.C.

Served as a speaker or a member of a speakers bureau for: Shire

**AUTHORS**

Alyssa N. Troeschel, MPH

Intramural Research Department, American Cancer Society, Atlanta, Georgia

Disclosure: Alyssa N. Troeschel, MPH, has disclosed no relevant financial relationships.

Corinne R. Leach, PhD, MPH, MS

Intramural Research Department, American Cancer Society, Atlanta, Georgia

Disclosure: Corinne R. Leach, PhD, MPH, MS, has disclosed no relevant financial relationships.

Kerem Shuval, PhD

Intramural Research Department, American Cancer Society, Atlanta, Georgia

Disclosure: Kerem Shuval, PhD, has disclosed no relevant financial relationships.

Kevin D. Stein, PhD

Intramural Research Department, American Cancer Society, Atlanta, Georgia

Disclosure: Kevin D. Stein, PhD, has disclosed no relevant financial relationships.

Alpa V. Patel, PhD

Intramural Research Department, American Cancer Society, Atlanta, Georgia

Disclosure: Alpa V. Patel, PhD, has disclosed no relevant financial relationships.

## Introduction

In 2016, the American Cancer Society (ACS) estimated there were approximately 15.5 million cancer survivors in the United States, and that population is projected to increase to 20.3 million in the next decade (1). Despite the increasing number of people surviving the disease, the transition from active cancer treatment to cancer survivorship (ie, *re-entry* phase) remains understudied, as are survivors’ health behaviors during this period (2). During re-entry, defined in this study as the first year following completion of primary adjuvant treatment, the focus shifts from diagnosis and treatment-related issues to long-term wellness and survivorship (3). This transition period may be used as a teachable moment to encourage health-promoting behaviors, including adherence to physical activity guidelines (3–5).

Among cancer survivors, physical activity has been associated with lower risk of all-cause mortality as well as improvements in physical functioning, anthropometric measures, health-related biomarkers, and health-related quality of life (6–9). As a result, the ACS guidelines for cancer survivors recommend at least 150 minutes of moderate-intensity or 75 minutes of vigorous-intensity aerobic activity each week (7).

Despite the health benefits of physical activity, studies suggest that most cancer survivors are insufficiently active, with adherence to physical activity guidelines ranging from 17% to 47% (9–15). Rates of adherence to guidelines and correlates of physical activity levels among survivors in the re-entry phase may differ from those further out from treatment and diagnosis, as suggested by previous studies indicating time since diagnosis is significantly associated with physical activity adherence levels (14,16).

Identifying characteristics of people at greater risk for nonadherence to physical activity guidelines may help inform best practices for clinicians to educate patients to increase physical activity adoption and adherence as well as identify people who may benefit the most from a physical activity–related intervention during the re-entry phase. Thus, we examine the prevalence of physical activity adherence among breast, colorectal, and prostate cancer survivors during re-entry in addition to medical and demographic characteristics associated with physical activity levels.

## Methods

### Data source and sample

Data were collected from the ACS’s National Cancer Survivor Transition Study (Transition Study) conducted in 2013. The Transition Study assessed perceived preparedness in cancer survivors who were transitioning out of active treatment into the re-entry phase of the cancer continuum. Participants were recruited from ACS’s constituent database, which is used to track and manage requests for information, services, and events sponsored by ACS throughout the United States. Participants were eligible for the study if they met the following criteria: 1) history of breast, colorectal or prostate cancer, 3 common cancers in the United States (17); 2) completed initial, curative treatment within the past 12 months (defined as the re-entry period in this study); 3) aged 18 years or older at diagnosis; 4) able to read and speak English; 5) confirmed US address; and 6) alive at the time of recruitment. Screeners to determine participant demographics and eligibility were sent to cancer survivors in the ACS constituent database. Of the 4,182 screeners returned, 1,670 survivors were eligible for the study. Primary reasons for ineligibility for the Transition Study included those in active treatment (n = 449) or those who completed primary curative treatment 13 months or more before completing the screener (n = 1,335). Those deemed eligible for the study were sent a survey and 1,211 respondents (72.5% of eligible participants) returned the completed questionnaire. Additional details regarding recruitment and study methodology were previously published (18). Respondents were further excluded from the current analytic sample if they 1) self-reported cancer recurrence (n = 12); 2) self-reported diagnosis of a new cancer (n = 11); 3) had missing data for the outcome of interest, physical activity (n = 22); or 4) had missing data for time since last treatment (n = 6). The final analytic sample consisted of 1,160 posttreatment cancer survivors (69.5% of eligible participants).

### Measures

#### Assessment of physical activity

The frequency, type, and intensity of physical activity was assessed via a questionnaire similar to that used in other large-scale epidemiologic cohort studies (eg, Cancer Prevention Study-II and 3, and the Nurses’ Health Study) (19,20). Specifically, participants were asked to estimate the number of hours per week spent on 11 moderate and vigorous activities during the past month. Frequency options were none, less than 1, 1 to 2, 3, 4 to 6, and 7 or more. To include an estimate of intensity, the *2011 Compendium of Physical Activities* was used to assign metabolic equivalent of task (MET) values to each activity (21). For activities with multiple MET values, a conservative approach was taken; that is, the lowest MET value was chosen. Activities were walking (3.5 MET), jogging (7.0 MET), running (9.8 MET), bicycling (4.4 MET), tennis/racquetball (6.0 MET), lap swimming (5.8 MET), aerobics class (eg, step, kickboxing, etc.) (5.5 MET), aerobic machines (eg, elliptical, rowing) (4.9 MET), sports activities (6.25 MET), dancing (5.4 MET), and other aerobic recreation (eg, hiking) (4.3 MET). The middle value of the frequency for each activity was multiplied by its assigned MET value (19). For example, if the respondent marked 4 to 6 hours per week, 5 was multiplied by the activity’s MET value and summed across all activities.

For each physical activity item, multiple marks were examined (n = 6). If the respondent marked multiple answers that were next to each other (eg, 1 to 2 hours/week and 3 hours/week), 1 response was randomly chosen. If the respondent marked multiple answers that were not next to each other (eg, 1 to 2 hours/week and 4 to 6 hours/week) then the activity was coded as missing. The ACS guidelines recommend engaging in at least 150 minutes of moderate-intensity physical activity per week, or an equivalent combination of moderate-intensity and vigorous-intensity aerobic physical activity, equating to approximately 8.75 MET hours per week (7,22). Physical activity was categorized in this analysis in relation to ACS guidelines resulting in 4 categories: 1) inactive (0 MET h/wk); 2) insufficiently active (0.01–8.74 MET h/wk); 3) 1 to less than 2 times the recommended levels (8.75–17.49 MET h/wk); and 4) 2 or more times the recommended levels (17.50 or more MET h/wk). The 17.50 or more MET hours per week category was used as the referent group.

#### Correlates

All variables included in the analyses were self-reported by the respondent. The following sociodemographic variables were considered for analysis: sex, race/ethnicity (non-Hispanic white, other), education (high school graduate or less, some college or more), and marital status (married, not married). Respondents categorized as married indicated that they were married or living in a marriage-like relationship, whereas not married respondents indicated they were single, separated, widowed or divorced.

The following clinical variables were considered: age at cancer diagnosis, body mass index (BMI), smoking status (never, current, former), cancer type (breast, colorectal, prostate), cancer stage (local, regional/lymph node involvement, distant/metastasis), receipt of surgery (yes, no) and receipt of chemotherapy (yes, no). BMI was calculated using the participant’s self-reported current height and weight. Additional clinical variables collected included time since the last cancer treatment and number of comorbidities. Time since last treatment was defined as the duration of time (months) from when the respondent last reported receiving surgery, chemotherapy, or radiation treatment to the time the respondent completed the survey. The number of comorbidities was derived from a standard checklist of 11 comorbid conditions (23): arthritis/rheumatism, glaucoma, emphysema/chronic bronchitis, high blood pressure, heart disease, circulation trouble in arms or legs, diabetes, stomach or intestinal problems, osteoporosis, chronic liver or kidney disease, and stroke. Conditions were summed and ranged from 0 to 11.

### Statistical analysis

Participant medico-demographic characteristics were descriptively summarized overall as well as at each level of physical activity. *P* values were calculated using simple multinomial regressions for each predictor variable independently. The 11 types of aerobic activities contributing to the overall physical activity level were summarized by cancer type and by level of physical activity. Stepwise multivariable multinomial regressions were then used to identify which medico-demographic characteristics were related to levels of physical activity. Variables of interest were based on prior literature (10,14,24–27): age, sex, race/ethnicity, education, marital status, smoking status, comorbid conditions, cancer type, cancer stage, BMI, months since last treatment, and receipt of surgery and chemotherapy. Age at cancer diagnosis, BMI, time since last treatment, and number of comorbidities were all modeled continuously. All predictor variables that reached the significance level of *P* < .20 were considered for the final multivariable model. To create the most parsimonious model, variables that did not reach significance level of *P* < .05 in the multivariable model were backward eliminated one by one. Because advanced cancer patients may be limited in their ability to be physically active, a sensitivity analysis that dropped people with cancer metastasis (n = 83) was conducted and yielded similar results. All statistical analyses were carried out by using SAS v9.4 (SAS Institute, Inc).

## Results

Approximately 8.1% of participants were inactive (n = 94), 34.1% were insufficiently active (n = 396), 24.3% were within 1 to less than 2 times the physical activity guidelines (n = 282), and 33.4% exceeded the recommended physical activity levels by 2 or more times (n = 388) (Table 1). Those exceeding guidelines by 2 or more times were, on average, younger (mean [standard deviation], 60.1 y [11.1]) than the other physical activity groups. Inactive people consisted of a higher proportion of women (9.6% of women were inactive compared with 6.1% of men) and people who were not married (10.9% versus 6.9% of those who were married were inactive). A higher proportion of those with a high school diploma or less were insufficiently active (41.9% versus 29.7% with some college education or more were insufficiently active). Multinomial regressions carried out for each predictor variable independently revealed that age, sex, race/ethnicity, education, marital status, smoking status, surgery, BMI, and number of comorbidities all varied (*P* < .20) by physical activity levels and were, therefore, considered in the multivariable model. There were no significant physical activity level differences by cancer type, months since last treatment, chemotherapy, or cancer stage.

**Table 1:** Medico-Demographic Characteristics of 1,160 Cancer Survivors During Re-entry^a^, by Level of Physical Activity, National Cancer Survivor Transition Study, 2013^b^

Characteristic	Total	Inactive^c^	Insufficient Activity^d^	Within Guidelines^e^	Exceeds Guidelines^f^	*P* Value^g^
**Overall**	1,160 (100)	94 (8.1)	396 (34.1)	282 (24.3)	388 (33.4)	NA
**Sex**						
Male	493 (42.5)	30 (6.1)	181 (36.7)	107 (21.7)	175 (35.5)	.02
Female	667 (57.5)	64 (9.6)	215 (32.2)	175 (26.2)	213 (31.9)	
**Race/ethnicity**						
Non-Hispanic white	974 (84.5)	71 (7.3)	341 (35.0)	243 (25.0)	319 (32.8)	.06
Other	179 (15.5)	22 (12.3)	54 (30.2)	37 (20.7)	66 (36.9)	
**Education**						
High school graduate or less	411 (35.6)	43 (10.5)	172 (41.9)	88 (21.4)	108 (26.3)	<.001
Some college or more	743 (64.4)	51 (6.9)	221 (29.7)	191 (25.7)	280 (37.7)	
**Marital status**						
Married	794 (68.9)	55 (6.9)	263 (33.1)	189 (23.8)	287 (36.2)	.02
Not married^h^	359 (31.1)	39 (10.9)	129 (35.9)	90 (25.1)	101 (28.1)	
**Smoking status**						
Nonsmoker	540 (50.4)	35 (6.5)	176 (32.6)	128 (23.7)	201 (37.2)	.19
Former smoker	428 (40.0)	37 (8.6)	145 (33.9)	110 (25.7)	136 (31.8)	
Current smoker	103 (9.6)	12 (11.7)	40 (38.8)	23 (22.3)	28 (27.2)	
**Cancer type**						
Breast	396 (34.1)	33 (8.3)	136 (34.3)	99 (25.0)	128 (32.3)	.26
Colorectal	423 (36.3)	41 (9.7)	129 (30.5)	105 (24.8)	148 (35.0)	
Prostate	341 (29.6)	20 (5.9)	131 (38.4)	78 (22.9)	112 (32.8)	
**Cancer stage**						
Local	706 (62.4)	56 (7.9)	251 (35.6)	173 (24.5)	226 (32.0)	.38
Regional or distant	426 (37.6)	33 (7.8)	135 (31.7)	101 (23.7)	157 (36.9)	
**Surgery**						
Yes	941 (81.3)	78 (8.3)	305 (32.4)	235 (25.0)	323 (34.3)	.09
No	217 (18.7)	16 (7.4)	90 (41.5)	46 (21.2)	65 (30.0)	
**Chemotherapy**						
Yes	716 (62.2)	66 (9.3)	233 (32.5)	174 (24.3)	243 (33.9)	.22
No	435 (37.8)	27 (6.2)	159 (36.6)	107 (24.6)	142 (32.6)	
**Continuous variables, mean (SD)**						
Age, y	62.2 (10.9)	62.8 (11.7)	63.5 (10.6)	63.0 (10.5)	60.1 (11.1)	<.001
Body mass index	29.0 (6.5)	30.3 (6.4)	30.4 (7.1)	28.1 (5.7)	28.0 (6.0)	<.001
No. of comorbidities	1.9 (1.6)	2.5 (1.7)	2.1 (1.6)	1.9 (1.6)	1.5 (1.5)	<.001
Time since treatment, months	7.8 (3.3)	7.4 (3.6)	7.7 (3.2)	7.9 (3.4)	7.9 (3.2)	.48

Abbreviation: MET, metabolic equivalent of task; NA, not applicable; SD, standard deviation.

a The transition from active cancer treatment into survivorship.

b All values are n (%) except where otherwise noted. All percentages presented above are valid percentages and do not include missing data. Percentages for missing data are as follows: age, 0.1%; sex, 0.0%; race/ethnicity, 0.6%; education, 0.5%; marital status, 0.6%; smoking status, 7.7%; cancer type, 0.0%; cancer stage, 2.4%; time since treatment, 0.0%; surgery, 0.2%; chemotherapy, 0.8%; body mass index, 10.0%; and number of comorbidities, 0.9%.

c 0 MET h/wk.

d 0.01–8.74 MET h/wk.

e 8.75–17.49 MET h/wk.

f 17.50 or more MET h/wk.

g *P* value is from the omnibus test of association from simple multinomial regression models.

h Not married includes people who are single, separated, divorced, or widowed.

The most common types of physical activity reported overall were walking (89.0%); bicycling (20.0%); elliptical, rowing, stair, or other aerobic machine (13.2%); and jogging (12.9%). Walking and bicycling remained the 2 most commonly reported activity types across both cancer type and level of physical activity (Table 2). The most common comorbidities included arthritis/rheumatism (46.1%), high blood pressure (45.6%), stomach or intestinal problems (24.5%) and circulation trouble in arms or legs (22.8%).

**Table 2:** Frequencies and Percentages for 11 Aerobic Activities Among 1,160 Cancer Survivors During Re-Entry^a^ Stratified by Cancer Type and Level of Physical Activity^b^, National Cancer Survivor Transition Study, 2013

Activity	Cancer Type, n (%)			Level of Physical Activity, n (%)		
	Breast	Prostate	Colorectal	Insufficient Activity^c^	Within Guidelines^d^	Exceeds Guidelines^e^
Walking (including treadmill)	346 (87.8)	371 (88.5)	309 (90.9)	371 (94.4)	278 (98.9)	377 (97.2)
Jogging (slower than 10 min per mile)	45 (11.4)	53 (12.7)	50 (14.8)	9 (2.3)	29 (10.3)	110 (28.6)
Running (10 min/mile or faster)	21 (5.3)	31 (7.4)	20 (5.9)	1 (0.3)	8 (2.9)	63 (16.3)
Bicycling (including stationary bike)	88 (22.3)	71 (16.9)	72 (21.2)	40 (10.2)	38 (13.6)	153 (39.5)
Tennis/racquetball	4 (1.0)	6 (1.4)	7 (2.1)	0 (0.0)	2 (0.7)	15 (3.9)
Lap swimming	19 (14.9)	12 (2.9)	13 (3.9)	4 (1.0)	6 (2.2)	34 (8.9)
Aerobics class (eg, step, kickboxing)	40 (10.3)	32 (7.6)	14 (4.2)	9 (2.3)	10 (3.6)	67 (17.7)
Elliptical, rowing, stair, or other aerobic machine	64 (16.3)	55 (13.2)	32 (9.5)	15 (3.8)	19 (6.8)	117 (30.6)
Sports activities (eg, soccer, basketball, baseball)	11 (2.8)	33 (7.9)	22 (6.5)	4 (1.0)	8 (2.9)	54 (14.3)
Dancing (eg, popular, folk, ballroom)	57 (14.6)	42 (10.1)	22 (6.5)	20 (5.1)	23 (8.2)	78 (20.6)
Other aerobic recreation (eg, golf without cart, hiking, skiing)	42 (10.9)	47 (11.3)	57 (16.9)	15 (3.8)	20 (7.3)	111 (29.2)

Abbreviation: MET, metabolic equivalent of task.

a The transition from active cancer treatment into survivorship.

b The 2011 Compendium of Physical Activities was used to assign MET values to each activity (21).

c 0.01–8.74 MET h/wk.

d 8.75–17.49 MET h/wk.

e 17.50 or more MET h/wk.

In the multivariable model, surgery, race/ethnicity, and smoking status were nonsignificant and therefore eliminated from the final model. The likelihood ratio χ^2^ test indicated the final model was significant (χ^2^ = 101.1, *P* < .001) and results are presented in Table 3. Multivariable analysis (Table 3) revealed that those who were inactive were significantly more likely to be women (adjusted odds ratio [AOR], 1.88; 95% confidence interval [CI], 1.10–3.23), not currently married (AOR, 1.81; 95% CI, 1.07–3.06), and have lower education levels (AOR, 2.02; 95% CI, 1.21–3.38) compared with those who exceeded the recommended levels by 2 or more times. In addition, a 1-unit increase in number of comorbidities was related to a 26% increase in odds of being inactive. Insufficiently active people were more likely to have lower education levels (AOR, 1.81; 95% CI, 1.30–2.52) and not be married (AOR, 1.43; 95% CI, 1.01–2.03) compared with those who exceeded the recommended levels by 2 or more times. A 1-year increase in age and a 1-unit increase in BMI were associated with 2% and 5% respective increases in odds of insufficient activity.

**Table 3:** Multivariate Multinomial Regression: Adjusted Odds Ratios (AORs) for Inactive, Insufficiently Active, and Within Guidelines, Compared With Exceeds Guidelines^a^, National Cancer Survivor Transition Study, 2013

Characteristic	Inactive^b^		Insufficiently Active^c^		Within Guidelines^d^	
	AOR (95% CI)	*P* Value	AOR (95% CI)	*P* Value	AOR (95% CI)	*P* Value
**Age, y**	1.02 (0.99–1.04)	.18	1.02 (1.01–1.04)	.004	1.03 (1.01–1.05)	<.001
**Sex**						
Female	1.88 (1.10–3.23)	.02	1.18 (0.84–1.64)	.34	1.70 (1.18–2.44)	.004
Male	1 [Reference]					
**Education**						
High school graduate or less	2.02 (1.21–3.38)	.01	1.81 (1.30–2.52)	<.001	1.15 (0.79–1.66)	.46
Some college or more	1 [Reference]					
**Marital status**						
Not married	1.81 (1.07–3.06)	.03	1.43 (1.01–2.03)	.046	1.38 (0.95–2.00)	.09
Married	1 [Reference]					
**No. of comorbidities**	1.26 (1.08–1.47)	.004	1.11 (1.00–1.24)	.06	1.05 (0.94–1.19)	.39
**Body mass index**	1.04 (1.00–1.08)	.04	1.05 (1.02–1.08)	<.001	1.00 (0.97–1.03)	.96

Abbreviations: AOR, adjusted odds ratio; CI, confidence interval; MET, metabolic equivalent of task.

a 17.50 or more MET h/wk.

b 0 MET h/wk.

c 0.01–8.74 MET h/wk.

d 8.75–17.49 MET h/wk.

## Discussion

To our knowledge, this study is one of the first to explore physical activity behavior among breast, prostate, and colorectal cancer survivors during the re-entry stage, a phase of the cancer continuum that can be used for behavior change. Our analysis reveals that approximately 58% of cancer survivors during re-entry met physical activity guidelines whereas approximately 8% were completely inactive. Women, those with lower education levels, those who were unmarried, and those with more comorbid conditions and higher BMIs were more likely to be inactive. Correlates of insufficient activity were similar, except sex and number of comorbidities were not significantly associated with insufficient activity and age was an additional correlate.

A higher proportion of cancer survivors in this study reported meeting guidelines, compared with similar US studies conducted among survivors with varying times since diagnoses with physical activity levels ranging from 20% to 47% (9,13–15). One US study among middle-aged cancer survivors reported 12% were inactive (14), which is slightly higher than our estimated 8%. The higher proportion of cancer survivors meeting physical activity guidelines in our study may be attributed to differences among survivors during the re-entry phase. These people, having recently completed treatment, may be more motivated to comply with the physical activity guidelines. Alternatively, our results may overestimate this proportion because of our sample demographics. The ACS Cancer Survivor Transition Study (18) largely consists of non-Hispanic whites recruited from ACS’s constituent database. Some participants could be regarded as “information seekers” (ie, contacted ACS for information or services) and are possibly more motivated to engage in cancer prevention behaviors than other samples. However, approximately 20% were recruited directly through physician fax referrals, which may mitigate the potential for such bias.

Several demographic factors were associated with level of physical activity among breast, colorectal, and prostate cancer survivors within 1 year of completing curative treatment. Female cancer survivors, in comparison with male cancer survivors, were more likely to be inactive. This finding is similar to overall population trends that illustrate men tend to be more physically active (28). Similar results were also found in other studies of cancer survivors such as the large, nationally representative Behavioral Risk Factor Surveillance System Survey, which reported male survivors were more likely to meet recommended levels of physical activity (14). In addition, in a study of colorectal cancer survivors, men reported higher levels of physical activity than women (27). People with lower education levels were significantly less likely to adhere to physical activity recommendations, which is consistent with results from other studies (10,11,14,24,25) as well as that in the overall adult population (28). Studies examining correlates of physical activity among colorectal cancer survivors found that more-educated survivors were also more physically active (11,25,26). Similar results were found among breast cancer survivors (10,24) and among all cancer types (10,14).

Older age was associated with being insufficiently active, but not with being inactive. These results may suggest that while older survivors are active, they are not active enough. However, only 8.1% of our study population (n = 94) were inactive, and this lack of association may be due to limited power among the inactive group. Regarding BMI, a previous study that also modeled BMI as continuous supports the notion that increasing BMI is associated with greater likelihood of being physically inactive among colorectal cancer survivors (27).

This study has several limitations and strengths. Physical activity was self-reported and is therefore subject to either overreporting or underreporting. Objective measurement of physical activity (eg, via accelerometers) alongside self-reported measurement is preferable (29), but was not available in the current data set as well as many other epidemiological studies. Additionally, while our sample was large, it primarily consisted of non-Hispanic whites recruited from ACS’s constituent database. Therefore, a more representative sample of cancer patients should be examined in future studies before generalizing findings. This study also is subject to self-selection bias because of survival differences (eg, inactive older adults are probably more likely not to survive and not to reach out for help via ACS channels). Lastly, our study is cross-sectional; therefore, temporality and causality cannot be inferred.

Nonetheless, our study is among the first to examine a large number of breast, colorectal, and prostate cancer survivors, 3 of the most common cancers in US adults, during the re-entry phase. Study findings suggest that 42% of breast, colorectal, and prostate cancer survivors in the re-entry phase are not meeting physical activity guidelines despite the numerous health benefits. Clinicians may be optimally positioned to deliver advice to cancer survivors on the importance of healthy lifestyle behaviors or provide further details about educational and/or training sessions about how to engage in safe and healthy behaviors as they near the end of curative treatment. This is an especially important discussion among older patients and patients with certain comorbidities, because they may be limited in their ability to be physically active and would need to discuss with their physician safe ways to engage in activity. While clinicians should educate all cancer survivors about their increased risk of recurrence, secondary cancers, comorbid conditions, and the benefits of physical activity, they may want to place special emphasis on educating women and those who are not married, are older, or have lower levels of education, comorbid conditions, or a higher BMI. These people might also benefit from targeted public health or clinical interventions or individual counseling. Previously conducted randomized, controlled trials suggest interventions offered to short-term cancer survivors during the re-entry phase can be effective (30). Future studies may want to examine physical activity adherence and its associated factors among a broad range of cancer survivors, as well as among specific cancer types, during re-entry to confirm our findings. Studies directly comparing physical activity levels of survivors during the re-entry phase to levels in more long-term survivors may help us better understand not only the differences between these 2 phases of the cancer continuum but also ways to increase the adoption and maintenance of physical activity throughout survivorship.

## Post-Test Information

To obtain credit, you should first read the journal article. After reading the article, you should be able to answer the following, related, multiple-choice questions. To complete the questions (with a minimum 75% passing score) and earn continuing medical education (CME) credit, please go to http://www.medscape.org/journal/pcd. Credit cannot be obtained for tests completed on paper, although you may use the worksheet below to keep a record of your answers.

You must be a registered user on http://www.medscape.org. If you are not registered on http://www.medscape.org, please click on the “Register” link on the right hand side of the website.

Only one answer is correct for each question. Once you successfully answer all post-test questions, you will be able to view and/or print your certificate. For questions regarding this activity, contact the accredited provider, CME@medscape.net. For technical assistance, contact CME@medscape.net. American Medical Association’s Physician’s Recognition Award (AMA PRA) credits are accepted in the US as evidence of participation in CME activities. For further information on this award, please go to https://www.ama-assn.org. The AMA has determined that physicians not licensed in the US who participate in this CME activity are eligible for *AMA PRA Category 1 Credits™*. Through agreements that the AMA has made with agencies in some countries, AMA PRA credit may be acceptable as evidence of participation in CME activities. If you are not licensed in the US, please complete the questions online, print the AMA PRA CME credit certificate, and present it to your national medical association for review.

## Post-Test Questions

### Study Title: Physical Activity in Cancer Survivors During “Re-Entry” Following Cancer Treatment

#### CME Questions

1. You are seeing a 62-year-old woman who is status-post partial colectomy with adjuvant chemotherapy for the treatment of stage II colon cancer. She is feeling better after some weakness and gastrointestinal complaints, and you provide counseling on physical activity during reentry. What should you consider regarding reentry and data on physical activity as you do so?
  - Reentry is defined as the first 3 months after primary adjuvant treatment for cancer
  - American Cancer Society (ACS) guidelines recommend at least 250 minutes of moderate-intensity physical activity per week among cancer survivors
  - ACS guidelines recommend at least 75 minutes of vigorous-intensity physical activity per week among cancer survivors
  - Approximately 70% of cancer survivors meet recommendation criteria for physical activity
2. The patient cites multiple barriers to becoming more physically active. What percentage of cancer survivors was either inactive or insufficiently active in the current study by Troeschel and colleagues?
  - 18%
  - 42%
  - 60%
  - 83%
3. Which of the following sociodemographic variables in the current study was most associated with physical inactivity?
  - Black race
  - Active cigarette smoking
  - Lower educational attainment
  - Male sex
4. Which of the following disease variables in the current study was most associated with physical inactivity?
  - A higher number of comorbidities
  - Colorectal cancer diagnosis
  - <3 months since the last cancer treatment
  - Higher cancer stage at the time of diagnosis
